# Accelerating Islamic charities in Indonesia: *zakat*, *sedekah* and the immediacy of social media

**DOI:** 10.1080/0967828X.2019.1691939

**Published:** 2019-12-04

**Authors:** Najib Kailani, Martin Slama

**Affiliations:** a School of Graduate Studies, State Islamic University Sunan Kalijaga, Yogyakarta, Indonesia; b Institute for Social Anthropology, Austrian Academy of Sciences, Vienna, Austria

**Keywords:** Development, Islam, social welfare, social media, Indonesia

## Abstract

The article describes the latest developments of Islamic charities and their role as major non-state actors in Indonesia's field of social welfare. It considers debates about the practices of Islamic charity in colonial and post-colonial times when *zakat* (obligatory almsgiving) was conceptualized as social welfare and as a tool for social justice or as an instrument to implement the post-colonial state's development goals. We examine the contemporary popularity of *sedekah* (voluntary almsgiving) among Indonesian middle-class Muslims and the rise of Islamic charities that specialize in *sedekah* programmes. These charities use social media to document their activities and to raise funds, and have changed the discourse of almsgiving. We argue that the temporal logic of acceleration increasingly informs the field of social welfare in Indonesia today. Islamic charities display their efficiency, transparency, and the material rewards that *sedekah* practices bring to donors. Today, Islamic charity is no longer primarily associated with social welfare and social justice but increasingly with economic gain. Accordingly, charities are concerned with accelerating their aid and its mediation, emphasizing immediacy in order to appeal to donors who demand a quick, unbureaucratic conversion of donations into concrete help, and an immediate material and spiritual return on their ‘investment’.

Indonesia represents a site where – under conditions set by different colonial and post-colonial regimes – state-driven and non-state-driven attempts to improve livelihoods have (dis)engaged with each other in multiple ways, resulting in different forms of (dis)entanglement. Among non-state initiatives, Islamic charities exemplify this often-ambiguous relationship between state and non-state actors. While these charities learned how to deal with state institutions during the colonial era, in independent Indonesia, especially since the establishment of Suharto's so-called New Order regime (1966–1998), Islamic charities have confronted a pervasive ideology of development. This article focuses on how ‘development’ resonates in Indonesia's Islamic charity discourses and, at the same time, it investigates particular transformations that occurred in the field of Islamic charity in the post-Suharto era. In this regard, the introduction of social media reinforced a trend towards the acceleration of charity work and its representation, which we regard as a crucial change in how Islamic charity is perceived and practised in Indonesia today, breaking with the teleological, future-oriented nature of ‘development’ (Fountain, Bush, and Feener 2015; Retsikas 2017) and emphasizing presence and immediacy instead. We further argue that this shift lies at the heart of today's stronger role of voluntary almsgiving (*sedekah*) in Islamic charity discourses, at the expense of discussions about obligatory almsgiving (*zakat*) that were concerned with the relationship between almsgiving, social welfare and social justice (Latief 2012; Fauzia 2013).

The genealogical investigation into Islamic charity discourses that this article provides forms the backdrop of an analysis that focusses on contemporary developments within Indonesia's field of Islamic charities. It considers the concurrence of political and technological changes in Indonesia, in other words, the end of the Suharto regime in 1998 and the rise of the internet and, in the late 2000s, the increasing use of social media and the ways in which they have been embraced in religious contexts (Slama 2017b). These changes have been accompanied by a repositioning of Islamic actors in relation to Indonesia's mediascape as well as by the introduction of discourses that stressed Islam's relevance for not only a pious but also a prosperous life (Rudnyckyj 2010; Hoesterey 2016; Kailani 2018). It is especially these discursive formations of economic interpretations of Islam – partly reflecting the globalization of neoliberal values such as self-reliance, efficiency and transparency – that have had a considerable impact on the evolution of Islamic charities over the last two decades in Indonesia (Retsikas 2017; for the broader South East Asian religious context see Koning and Njoto-Feillard 2017; as well as Atia 2013; Tugãl 2017; Mittermaier 2019 and Muehlebach 2013, for illuminating examples from Egypt, Turkey and Italy respectively).

Taking account of the prominence of *sedekah* in these discursive formations, however, is not to say that *zakat* is now considered a less important Islamic practice – it is of course still regarded as an obligation – or that *zakat* management organizations have become less active. Yet the interpretations of *sedekah* that have spread widely in post-Suharto Indonesia enabled Muslims, particularly middle- and upper-class Muslims, to combine their quest for piety and prosperity in new ways. At the same time, questions of social justice that were associated with *zakat* by some Islamic figures during the New Order have receded into the background. Today, *sedekah* is conceptualized as a way to gain wealth in this life, as it is grounded in the belief that God will give back more than one has donated and, most significantly, that this will happen shortly after one has done so. *Sedekah* is thus less related to the hereafter than to the here and now, which leads us to consider *sedekah* – and indeed Islamic charities as such (Pall 2015) – as a phenomenon of the contemporary, globalizing world.

Many regions within Indonesia, especially the urban areas on the central island of Java where most of Indonesia's middle-class *sedekah* donors live today, have become part of what Thomas Hylland Eriksen (2016, 469) describes as ‘a world of high-speed modernity’, in which acceleration has become a guiding principle in many spheres of life. We consider the shift of emphasis from *zakat* to *sedekah* as representing a theological expression of this trend, since it goes hand in hand with an attempt to speed up the process of receiving rewards from God for one's pious deeds. Ideally, *sedekah* generates an instant, immediate return that enhances not only the spiritual but also the material well-being of the donor. This acceleration of exchanges between God and the believer point to the new temporalities that have been introduced into Islamic charity, such as we can observe in Eriksen's high-speed modernity more generally, and that need to be facilitated by new media. In the field of Islamic charities in particular, social media function as the medium of acceleration as well as immediacy, another key concept that we briefly discuss in this introduction.

Today, Islamic charities use social media to document their activities and to attract funding. In doing so, they project an image of real-time response to the people in need and of a quick allocation of the funds that they have received. Social media mediate not only the good deeds of the charities and the careful and transparent handling of financial issues but also this sense of immediacy that lies at the heart of the concept of *sedekah* as it is interpreted in Indonesia today. When immediate help is seen as resulting in immediate returns, the same temporal logic is at work here. However, this is not only a question of temporality. The immediacy of media, as Patrick Eisenlohr (2011, 44) has argued, refers to ‘the tendency of media to disappear in the act of mediation’, which – in the context of religion in particular – means that people ‘search for new media affording seemingly more “direct” and “immediate” relationships to others, political and religious institutions, or God’ (Eisenlohr 2011, 44).

This more direct relationship to the mundane and the divine is what Indonesia's new Islamic charities suggest they provide on their social media platforms and through their *sedekah* economy. They thus also seem to have found a way to anticipate what Erica Bornstein (2009) has called ‘the impulse of philanthropy’, that is, the desire to end suffering through immediate help. Indeed, they like to emphasize their unbureaucratic and efficient responses to humanitarian crises. A series of natural disasters in post-Suharto Indonesia, such as the devastating tsunami of 2004 and several deadly earthquakes and floods, have raised awareness of the importance of being able to help as quickly as possible.^1^ Acceleration in this sense, that is, with regard to the ability to launch humanitarian operations without delays, thus has also become a challenge for Islamic charities that today usually maintain a quick response unit (*Aksi Cepat Tanggap*) specialized in dealing with natural disasters (Latief 2013, 186). Before we describe phenomena of acceleration in the field of Islamic charities in contemporary Indonesia, we first examine how Islamic charity was conceptualized prior to the rise of high-speed modernity.

## A brief genealogy of Islamic charity discourses in Indonesia

Charity in Islam originates from the concept of *zakat,* an annual obligatory act of worship to distribute wealth among the poor and indigent. As such, *zakat* is a means to purify Muslims’ wealth. It is divided into two broad categories: *zakat maal* and *al-fitr*. *Zakat maal* accounts for 2.5% of a Muslim's wealth and can be paid anytime; meanwhile, *zakat al-fitr* is only conducted at the end of Ramadan, that is before the Eid of Fitr (celebration day at the end of Ramadan), by providing food to the poor and needy. In the Qur’an, the term *zakat* overlaps with *sadaqa* or *sedekah* (Al-Qur’an 9:60). It refers to benevolence and rectitude. However, the main difference between them is that *zakat* is compulsory for Muslims, while *sedekah* is a voluntary act. In addition, if *zakat* signifies worship through distribution of wealth, *sedekah* has a broader connotation, which refers to not only material but also nonmaterial ways of giving, including kind words, friendly smiles and passing on knowledge. In addition to *zakat* and *sadaqa*, *waqf* (endowment) is also a part of Islamic charity (Benthall 1999, 2012; Kochuyt 2009; Atia 2013; Singer 2013).

In late colonial Indonesia, during the first half of the twentieth century, the Qur’anic injunction of *zakat* was interpreted not merely as compulsory worship but pivotally as an instrument for social welfare. This idea was particularly pioneered by Muhammadiyah, Indonesia's largest reformist Muslim organization, comprising a critique of the existing practice of *zakat* collection and distribution (Fauzia 2017a; Nakamura 1993, 90). Traditionally, *zakat* was collected by *modin –* people who are committed to perform *adzan* (the call for prayer) and prayer at mosques – and was distributed mostly among the Muslim congregation of the mosque (Fauzia 2013, 158). However, Muhammadiyah suggested that *zakat* should be managed by professionals and distributed to the poor and indigent. In order to spread this idea, Muhammadiyah referred to the Qur’anic verse al-Maun^2^ to justify and energize its social activities by establishing orphanages, hospitals and modern educational institutions in urban Indonesia (Latief 2010a, 126–127). Traditional *zakat* practices and the reformist critique they evoked dominated the inner-Islamic discourse for decades.

In independent Indonesia, however, especially in the 1980s, when Indonesia's Muslim middle class started to expand, *zakat* and other forms of Islamic charity were articulated as not merely an instrument for social welfare but also for social justice (Retsikas 2014, 344; Fauzia 2017b). This change in discourse cannot be separated from the responses of Indonesian Muslims to New Order developmentalism. The advocators of *zakat* as a form of social welfare were Muslim scholars who supported New Order developmentalism, whereas the promoters of social justice often had an NGO background and voiced their concerns about the negative impacts of New Order development projects, especially with regard to the widening gap between the rich and the poor. In the following section, we will briefly analyse the advocators of the two discourses of *zakat* and highlight their ideas in order to be able to contrast them with post-Suharto developments.

Although Suharto restricted expressions of political Islam in public, he agreed with the idea of promoting Islamic piety among Indonesian Muslims, especially when Islamic ideas could be mobilized to support New Order development programmes. Recognizing the prospect of *zakat* as an alternative form of social welfare, Suharto demonstrated his support for *zakat* by establishing a state institution to collect *zakat,* named *Badan Amil Zakat* (BAZ; State-based Zakat Agency). Amelia Fauzia argues (2013, 189) that Suharto's approval of functionalizing *zakat* as social welfare indicated his efforts to integrate Islamic ideas into his development ideology. Under such political conditions, Indonesian Muslim scholars actively engaged in national development through re-interpreting Islamic teachings. According to Howard Federspiel (1998), Muslim scholars discussed three major Islamic concepts in relation to national economic development, among which sharing wealth through religious tax (*zakat*) was one, in addition to viewing humans as stewards of God and as having a fear of God (*taqwa*). These three Islamic concepts were interpreted, articulated and mobilized in order to support New Order development projects.

The new interpretation and articulation of *zakat* during the New Order is linked to the ‘Islamic renewal movement’ (*gerakan pembaharuan Islam*) which emerged in the late 1970s. This movement was supported by prominent Muslim figures who emphasized the need to contextualize, re-articulate and modernize Islamic teachings through *ijtihad* (reasoned interpretation) of the Qur’an and Hadith (words and deeds of the Prophet Muhammad), as well as the classical literature written by the *ulama* (Islamic scholars). One example of the renowned figures who pioneered the contextualization of *zakat* in Indonesia was M. Hasbi As-Shiddieqy, a professor of Islamic studies at State Islamic University Sunan Kalijaga, Yogyakarta, and a member of Muhammadiyah. He proposed to create an Indonesian version of Islamic law or *fikih Indonesia* considering the local context of Indonesian Islam (Fauzia 2013, 182; Feener 2007, 65–66). With regard to *zakat* in particular, As-Shiddieqy suggested the need to re-interpret the notion of *Al-Maal Al-Mustafad* (wages earned regularly by an individual) as the subject of *zakat* if one receives wages for more than one year (*hawl*) (Latief 2012, 91). According to Hilman Latief (2012, 90–91), As-Shiddieqy's argument regarding *Al-Maal Al-Mustafad* has contributed to shaping the idea of *zakat profesi* (*zakat* on regular income) advocated by the Muhammadiyah leader Amien Rais in the 1980s (see below).

As a closer look reveals, the ‘Islamic renewal movement’ went hand in hand with Suharto's New Order development agenda since the advocators were mostly highly ranked officers in Suharto's cabinets such as Mukti Ali and Munawwir Syadzali, who both served as minister of religious affairs (from 1973 to 1978 and from 1983 to 1993 respectively). During the New Order, Muslim intellectuals also aligned Islam with national development projects by promoting ‘development dakwah’ (*dakwah pembangunan*), which can be translated as proselytization through development (Federspiel 1991). The ‘development *dakwah*’ was marshalled by the Ministry of Religious Affairs and supported by Muslim religious leaders and scholars. They emphasized *dakwah bil-hal* (Islamic propagation by deeds) in addition to *dakwah bil-lisan* (Islamic propagation by words), such as functionalizing *zakat* as social welfare (Meuleman 2011; Sakai 2014).

Ronald Lukens-Bull (1996, 266–277) has demonstrated that Muslim intellectuals have also held different opinions about New Order development projects, as some of them mobilized Islamic ethics to address the negative impacts of the New Order's development policies, including growing social inequality. These Muslim intellectuals extended the notion of *zakat* to the sphere of social justice and thus transcended the limitations that are inherent in views that associate it with social welfare. They sought to marry Islamic values with Latin American liberation theology as formulated by Paulo Freire. Important proponents of this model include Yusuf Hasyim, Dawam Rahardjo, Muslim Abdurrahman, Mansour Faqih and Abdurrahman Wahid. They introduced the notion of ‘transformative Islam’ or ‘Islamic liberation theology’ to respond to the issue of poverty. However, in theological terms, these scholars have not paid particular attention to *zakat* but to other issues including community development and economic initiatives.

Amin Rais and Masdar Farid Mas’udi are two Muslim scholars who notably re-interpreted *zakat*. Amin Rais received his PhD from the University of Chicago with a thesis about the Muslim Brotherhood and later became chair of Muhammadiyah (1995–2000) and active in politics in post-Suharto Indonesia. In the late 1980s, Rais proposed the idea of ‘*zakat profesi’* (*zakat* on regular income). This notion, as examined by Kostas Retsikas (2014, 345), was based on the argument that regular income (such as a salary) should be interpreted as wealth that is subject to *zakat*. Thus, *zakat profesi* reveals the changing social-economic contour of society from agriculture to urban-industrial forms of income. Consequently, the percentage of *zakat profesi* does not necessarily amount to 2.5% (one-fortieth) of one's income, since one should consider income disparities between professions. Rais, who was inspired by Sayyid Qutb's ideas on social justice, argued that *zakat profesi* significantly reflects the idea of social justice in Islam.

Such progressive approaches to *zakat* are not unique to Muslims with a reformist background but can also be found among Muslims with a traditionalist one. Masdar Farid Mas’udi, from the traditionalist camp, is a central figure when it comes to new interpretations and articulations of *zakat* as social welfare and social justice. He graduated from Pesantren Tegalrejo Magelang, Central Java, and studied at the State Islamic Institute Sunan Kalijaga in Yogyakarta (later renamed State Islamic University). In the 1980s, he was an activist at the Centre for the Study of *Pesantren* and Society (*Pusat Pengkajian Pesantren dan Masyarakat*, P3M) together with Abdurrahman Wahid, and initiated economic community development programmes by relying on the *pesantren* network of Nahdlatul Ulama (NU), Indonesia's biggest Islamic organization. In his 1991 book entitled *Religion of Justice: A Treatise on Zakat (Tax) in Islam* (*Agama Keadilan: Risalah Zakat (Pajak) dalam Islam*), he suggested that *zakat* could be an instrument of social welfare and social justice if it is understood as a modern-day tax. Whereas Amin Rais located his reasoning on the importance of *zakat profesi* in social analysis, Mas’udi referred to Islamic history and classical Islamic literature to formulate his argument about *zakat* as tax (Retsikas 2014; see also Feener 2007; Fauzia 2013).

In addition to these various attempts to conceptualize *zakat* as an instrument for social welfare and social justice, there have also been initiatives to translate these ideas into practice. The pioneer in this regard is the Dompet Dhuafa Foundation, founded in 1993 by journalists of the Islamic newspaper *Republika*. Dompet Dhuafa is a private philanthropic organization aimed at collecting funds from Indonesian Muslims and distributing them to the needy and indigent. In contrast to the traditional model of Islamic charity distribution, Dompet Dhuafa uses the donations for particular activities such as establishing health clinics for the poor and economic community development programmes (Latief 2010b, 517–521; Sakai 2010, 2012). Dompet Dhuafa's model of Islamic charity has contributed to the booming activities of Islamic charities in the post-Suharto era.

## Economized Islam and forms of Islamic charity in post-Suharto Indonesia

Although we saw a rapprochement between the regime and Islamic forces in the late New Order – which also comprised the field of Islamic charities – as well as intellectual attempts to reconceptualize *zakat* as a tax that should be used to enhance social welfare and establish social justice, there remained considerable scepticism concerning the question whether state institutions should administer these funds. One should not forget that the New Order was brought down by protests against KKN, as it was called, standing for corruption, collusion and nepotism (*korupsi*, *kolusi* and *nepotisme*), with the foundations (*yayasan*) run by the Suharto family attracting particular criticism. The early post-Suharto years were thus characterized by a high level of awareness regarding how funds can be misused and mismanaged by the state and its representatives who can seize state institutions for their personal benefit.^3^ In such a political climate, it does not come as a surprise that Muslims increasingly turned to Islamic non-state actors for their charity activities.

At the same time, new players emerged in Indonesia's Islamic field that were not only concerned with Muslims’ *zakat* obligations and their engagement in charity, but also with the economic outlook of the Muslim community at large. Consequently, the discourse on delivering welfare services and social justice was complemented – and partly superseded – by questions of how to generate wealth as a good Indonesian Muslim. As Daromir Rudnyckyj (2010, 126) has shown, these discourses did not replicate but also did not completely break with New Order developmentalism: ‘The very same modernist logic that had guided the project of Indonesian development was still at work in initiatives to develop faith’. His research about an Indonesian company led by a charismatic Muslim that dominated the market for motivational seminars in the 2000s indicates how Islam was reinterpreted as a guide to become a successful employee. Acting professionally in one's job as a modern Muslim was recast as a central part of one's piety. According to these motivators, to worship God and to work became two almost indistinguishable activities (see also Rudnyckyj 2008).

Pushed to the extreme, this programme of an Islamic work ethic became irreconcilable with earlier approaches that counted on Islamic charity to enhance the welfare of Muslims. The logic that being a good Muslim means to be a hard worker and thus economically successful is of course less appealing to Indonesian Muslims who work a lot but nevertheless receive only a small income than to members of the middle class. Only for the latter does work plus piety correspond to realistic chances for economic prosperity (Rudnyckyj 2015). And only in such a middle-class social environment it is intelligible why management jargon is so easily used to make sense of *zakat*, such as referring to it as ‘strategic collaboration’ (Rudnyckyj 2010, 91).

The emphasis on economic success – or the promise of a more prosperous future that theoretically can also be seized by those still poor in the present (Retsikas 2017) – in connection with Islamic charity is indeed a central premise of the discursive shift that has occurred in the post-Suharto era and that has been further propelled by a range of non-state actors in Indonesia's field of Islam. In this regard, the rise of Islamic preachers in post-Suharto Indonesia has to be considered as a significant development. In the 2000s, mainly due to their regular appearance on private TV stations, some Islamic preachers reached celebrity status and were skilful enough to convert their newly acquired cultural capital into economic gain. Furthermore, some of these wealthy preachers made economic success into a topic of their teachings, presenting themselves as role models for how piety can lead to prosperity. For example, Aa Gym (elder brother Gym), the most popular preacher in the mid-2000s, depicted the Prophet Muhammad as a *professional* and *entrepreneur,* and suggested that Muslims should join him in following the shining example of the Prophet. Aa Gym developed an entrepreneur-training programme that was – thanks to his popularity – also booked by Indonesian companies and banks to educate their employees. As analysed by James Hoesterey (2016, 113–117), this programme fused Western individualist self-help guides with Islamic economic ethics and emphasized Islamic charity practices as an integral part of an entrepreneurial Muslim's activities. Engaging in charity is regarded as crucial – and this is indeed central to our analysis as well – because it is believed to pay off not only in spiritual terms in the hereafter but also in material terms in this life.

Moreover, as Latief (2017) has pointed out, the collection of *zakat* by private players was accompanied by the introduction of management techniques that could be used for various strategic goals. For example, the *zakat* foundation of the preacher Aa Gym invested funds in a childcare training programme for young rural women with a lower-class background. The training comprises not only the subject of childcare per se, but also lessons in Islamic piety, suggesting that these childcare workers can also contribute to the Islamic education of the children they take care of. Investing *zakat* funds in such activities means to convert *zakat* into *dakwah* (proselytization) among rural lower-class women by transforming them into a skilled Islamic labour force that fits the expectations of their Muslim middle-class employers. This represents one example of how broad the field of Islamic charity has become within the country, generating ‘an unprecedented level of social activism within Muslim communities in Indonesia’ (Latief 2013, 191). Today, these activities are not restricted to *zakat* funds but can also include other sources such as Islamic savings and credit cooperatives that seek to provide capital to small-scale entrepreneurs, and thus to contribute to the establishment of a more socially just economy in Indonesia (Sakai 2014).

This trend towards an intensifying culture of giving in post-Suharto Indonesia was also anticipated by Indonesia's big Islamic organizations. In 2002, Muhammadiyah established its national *zakat* foundation LAZISMU, which was followed in 2004 by Nahdlatul Ulama launching NU Care LAZISNU. Although both organizations have an established membership, they could not afford to ignore the significant transformations in the field of digital technology and its social and economic consequences in Indonesia (Pangestu and Dewi 2017). They also adopted the model of employing social media platforms to promote Islamic charity and call for donations, the focus of our next section. Most importantly, they – as many other Islamic philanthropic organizations – started to utilize online banking and digital apps that made making and receiving donations much less complicated. Applications like Go Pay or OVO, to mention the perhaps most popular money transaction platforms in Indonesia today, enable making donations in a few seconds on one's smartphone, whereas previously Muslims had to personally visit orphanages or mosques to offer donations. We shall keep these technological changes in handling finances and their accelerating effects in mind when we discuss social media practices of giving in more detail.

## Islamic charities and social media practices: the rise of *sedekah*

In addition to new interpretations of *zakat* and novel ways of using *zakat* funds, the discourse of Islamic charity in post-Suharto Indonesia is epitomized by a heavy emphasis on the virtue of *sedekah* (voluntary almsgiving), especially the multiple material rewards one can receive from God when performing *sedekah*, rather than focusing on its social welfare and justice aspects. This practice is advocated by new Islamic authorities including celebrity preachers and Muslim businessmen who adhere to an economic theology of performing voluntary alms by promoting the ‘mathematic of voluntary almsgiving’ (*matematika sedekah*), using various platforms including print publications, films and especially social media (for the role of social media in Indonesia's preacher economy see Slama 2017a). Their *sedekah* discourse of seeking multiple material rewards is tailored to Indonesia's growing Muslim middle class that aspires to become pious and prosperous at the same time. Performing *sedekah* demonstrates volunteerism, which is widely recognized as part of the lifestyle of the middle and upper classes and as an expression and articulation of their public piety (Atia 2013, 12). Moreover, the popular practice of performing almsgiving among the urban Muslim middle class has recently been identified as ‘the rise of the giving community’ (*kemunculan komunitas memberi*) in Indonesia (Yuswohady, Fatahilah, and Ali 2017, 223). The following account will highlight two Muslim figures who have contributed to the new articulation and practice of Islamic charity in post-Suharto Indonesia, namely the influential national figure Yusuf Mansur and Saptuari Sugiharto, who became popular on a more regional level.

The pivotal figure behind the circulation of the economic theology of performing voluntary almsgiving among Indonesian Muslims is Yusuf Mansur. He is one of the most popular Muslim celebrity preachers in contemporary Indonesia, and centres his sermons on two key themes, namely *Kun Fayakuun* (literally ‘be, and it is’) and ‘the miracle of voluntary giving’ (*keajaiban sedekah*). The *Kun Fayakuun* is taken from the Qur’an in chapter *Yaasin,* which highlights that anything can occur if God wishes it to happen. Moreover, ‘the miracle of voluntary almsgiving’ portrays the virtue of performing *sedekah*, including the multiple rewards that givers will receive from God. Within the larger picture of Islamic charities in Indonesia, Yusuf Mansur is a pioneering figure who epitomizes the celebrity preacher-cum-charity organizer, demonstrating that not only Indonesia's big Islamic organizations or Islamic media corporations can be successful in this field. Moreover, given the primary focus on *zakat* by the established players, with *sedekah* Yusuf Mansur found a publicly less explored topic, a market niche that he was able to expand and fill in unprecedented ways.

Yusuf Mansur's main message about *sedekah* is that it is not only a means to distribute wealth to the needy but it is also pivotally a method that donors can use to seek spiritual and material wealth for themselves. To reinforce this idea, Mansur wrote a book entitled *An Introduction to the Miracle of Giving* (Mansur 2011), outlining his economic theology, which he calls ‘the mathematics of voluntary almsgiving’. In it, Mansur refers to the basic Islamic scriptures, such as verse 160, chapter 6, of the Qur’an that says, ‘He that doeth good shall have ten times as much to his credit: He that doeth evil shall only be recompensed according to his evil: no wrong shall be done unto (any of) them’ (6:160, *Al-Ana’m*) (Ali 1960, 194). He also refers to verse 261 in chapter 2 that reads, ‘The parable of those who spend their substance in the way of Allah is that of a grain of corn: it groweth seven ears, and each ear hath a hundred grains. Allah giveth manifold increase to whom He pleaseth: And Allah careth for all and He knoweth all things’ (2:261, *Al-Baqarah*) (Ali 1960, 65). Additionally, Mansur frequently cites a Hadith from the Prophet Muhammad saying ‘Wealth never decreases because of voluntary almsgiving but on the contrary increases’, which the Prophet Muhammad emphasized three times (Kailani 2015, 70).

Yusuf Mansur uses various channels to promote his economic theology, including religious television programs and social media, in order to reach wider audiences. For example, he spreads his sermons through a regular television program called *Keajaiban sedekah* (the miracle of voluntary almsgiving) that later changed its name to *Nikmatnya Sedekah* (the pleasure of voluntary almsgiving)*.* In addition, Mansur is active in circulating his ‘mathematics of voluntary almsgiving’ on social media. With more than seven million followers on Facebook and more than three million on Twitter, Mansur is recognized as one of Indonesia's social media celebrities.^4^ Consequently, it is not surprising that his economic theology has become widely popular among Indonesian Muslims.

In order to facilitate donations from his followers, Yusuf Mansur founded the Cultivation Programme for Memorizing the Qur’an (Program Pembibitan Penghapal Al-Qur’an Daarul Qur’an [PPPA Daarul Qur’an]) in 2007, a foundation that collects and distributes voluntary alms. In contrast to existing charity organizations in Indonesia, such as Dompet Dhuafa, PPPA Daarul Qur’an concentrates its activities on Qur’anic matters, such as establishing Islamic boarding schools for memorizing the Qur’an, providing scholarships to the needy for the study the Qur’an and to support teachers who are involved in the teaching of the Qur’an. In other words, PPPA Daarul Qur’an is a voluntary almsgiving institution for the fan community of Yusuf Mansur.^5^

One of Yusuf Mansur's followers who became an enthusiastic advocator of ‘the mathematics of voluntary almsgiving’ is Saptuari Sugiharto. Unlike Mansur, who is well known as a preacher, Saptuari is a businessman who runs a creative company called *Kedai Digital* (Digital Outlet), which creates digitally imprinted merchandise, from mugs to t-shirts. One day Saptuari watched Yusuf Mansur's television sermon and became interested in the message that *sedekah* would prevent misfortune, increase livelihood and heal illnesses. Saptuari realized that *sedekah* could also have a positive impact on the development of his business. In fact, his business expanded to many regions of Java, and he now owns forty-two Kedai Digital outlets through profit-sharing arrangements. He has also extended his business to include restaurants offering meatballs and satay as speciality dishes.

Saptuari actively circulates the economic theology of *sedekah* through social media. As of October 2019, his Twitter account @Saptuari was followed by 133,000 people. As early as 2011, his tweets were awarded as being the most inspirational by the platform *Pesta Blogger ON-OFF*, an annual festival of Indonesian blog writers, Twitter and Facebook users, supported by the Ministry of Communication and Information Technology, the Ministry of Research Technology and Higher Education, and the Ministry of Tourism and Creative Economy. In his tweets, Saptuari frequently addresses topics that are related to business and *sedekah* practices. The popularity of his tweets has also attracted a publisher, who adapted them into a book entitled *Sadistic Tweets that Give You the Creeps! A Collection of Inspiring Tweets that Make You More Creative* (Sugiharto and Beatrix 2012). Moreover, Saptuari promotes the economic theology of *sedekah* through his blogspot.^6^ The essays published there were later assembled in a book entitled *Beautiful Notes for God* (Sugiharto 2014). In this book, he explores how religious commitment, especially performing voluntary almsgiving, leads to a successful life.

In 2011, Saptuari and several Muslim business people in Yogyakarta founded a charity initiative named *Sedekah Rombongan* (Togetherness in Performing Voluntary Almsgiving). *Sedekah Rombongan* is an initiative that aims at helping the poor and needy who have serious illnesses. The idea of starting *Sedekah Rombongan* was triggered by Saptuari's blogs, where he has written on various topics, from entrepreneurship to inspirational stories. Saptuari explained that the term *rombongan* refers to the philosophy of the ant. While ants are considered to be small creatures, they are able to carry food by virtue of ‘togetherness’ or collective effort.^7^ In order to promote this idea, Saptuari created the hashtag #SedekahRombongan on Twitter, inviting a wide section of the population to become involved in and contribute to this benevolent initiative (Kailani 2015, 129).^8^ By adopting and propagating the concept of *sedekah*, just as his great role model Yusuf Mansur had done, Saptuari's *Sedekah Rombongan* established itself in the field of Islamic charities in Yogyakarta, where the charities of Indonesia's big Islamic organizations also have a strong presence, especially Muhammadiyah, which was founded in the city.

*Sedekah Rombongan* promotes economic theology as a way of mobilizing donations through social media. He regularly posts verses from the Qur’an that make mention of the virtues of *sedekah*. He also publishes success stories of people who received material rewards as a result of giving *sedekah*. Saptuari claims that *Sedekah Rombongan* is a new model of Islamic charity called *Sedekah Jalanan* (Street Almsgiving Endeavour). In its vision, *Sedekah Rombongan* states:

This is a street voluntary almsgiving endeavour. It is about people who cannot afford to buy medicines and food … It is about baby formula and meals … and also about students who cannot pay their tuition fees … In addition, it is also about the building of orphanages, Islamic boarding schools, and homestays for the poor that need to be refurbished and extended …  #SedekahRombongan delivers provisions from the sky (God) in an uncomplicated, easy and unconvoluted way.^9^

The advocates of *Sedekah Rombongan* explain the term *Sedekah Jalanan* as signifying their immediate action to help the poor and needy without any procedural and complicated assessments. If they receive information about those in need who cannot afford to pay for medical treatments, they will immediately go visit them and gather information about the patient. In addition, the term also refers to their commitment to deliver 100% of donations to the poor and needy. Saptuari emphasized that ‘We do not take a 10% administration fee (*amil*) from the donations, we do not get any salaries from this activity, we do not spend money for advertisements, and we do not need to rent an office. Our office is only a website and Twitter account and we utilize them to promote *Sedekah Rombongan*’.^10^

In order to assess the beneficiaries, *Sedekah Rombongan* does not have strict criteria. In relation to those who have a serious illness, they will check their medical reports and consult with the doctor in the hospital. If the doctor says that there is a reasonable chance of recovery for the sick, *Sedekah Rombongan* will take the sick to the hospital and cover all of their expenses during the treatment period. If a doctor declares that the sickness is acute and has little chance of recovery, *Sedekah Rombongan* will only provide a donation to support the sick.^11^

The simple process of assessment conducted by *Sedekah Rombongan* volunteers has become their primary means of action in helping the poor. They explained that helping the poor and needy needs to be done immediately in order to release them from their suffering. Erica Bornstein (2009) explains that the essence of philanthropy is originally impulsive and spontaneous. It is associated with an immediate desire to relieve a person of their suffering and distress. However, when charity is projected as a long-term project in the form of poverty alleviation, or regulated through moral judgments, philanthropy becomes a rational and instrumental action. Consequently, the rational action of philanthropy has replaced the impulsive and spontaneous action of philanthropy. By describing its initiative as a ‘street endeavour’ (*Sedekah Jalanan*), *Sedekah Rombongan* attempts to reintroduce the essence of a charitable act, namely ‘a kindly desire to end misery and suffering’ (Bornstein 2009, 623). It deliberately challenges institutionalized charity that works on strict procedures and criteria which reduce the immediacy and compassionate feeling to redress the suffering of the unfortunate other.

Social media have also enabled wide support and responses. *Sedekah Rombongan* volunteers believe their initiatives have become popular due to the attention they have received from established Muslim business people. For example, Yusuf Mansur, who previously had not personally known Saptuari and his colleagues, gave support to *Sedekah Rombongan* through the medium of Twitter:

We not only need honest and trusted people to call and distribute voluntary alms but we urgently need people who donate these alms with love and passion. I have found them at @saptuari and with my #SedekahRombongan colleagues. I have shed tears when I realised that feelings of compassion have started to decline in this country. However, I am optimistic through what I have seen that the senses will be back for one aim: Allah, everything for Him.^12^

Such tweets of prominent figures like Yusuf Mansur certainly boosted the popularity of *Sedekah Rombongan*. Yet the centrality of social media for new charities like *Sedekah Rombongan* is not restricted to their efforts to become known among potential donors. In fact, social media have become crucial for their daily operation. For example, *Sedekah Rombongan* reports regularly about their charitable acts on Twitter and Facebook, showing pictures of the patients that have received help (Kailani 2015, 139–140; see also Azis 2018). Its Twitter account leaves the impression of providing almost real-time news of its activities. Social media like Twitter are used to demonstrate how the charity's ideology of immediacy, transparency and unbureaucratic help is translated into concrete action. The real-time communication facilitated by Twitter thus constitutes the perfect medium to represent instant help. Following Eisenlohr (2011), the medium itself disappears in this process, when people do not realize anymore that these events are mediated.

The idea of transparency in this context, that a medium makes something visible that otherwise cannot be seen, applies not only to the concrete good deeds of the helpers but also to the finances that form the material basis of their charitable practice. It is quite common that receipts for items such as food, medicine or hospital bills are posted on social media stressing accountability in financial terms. Sometimes, donations are made public (e.g. the identity of the recipient, and in some cases also the donor, is disclosed), if the donation is meant for a particular person whose case was handled by the Islamic charity. Some donors even report their *sedekah* activities, especially when they result in an increase of their wealth, as asserted by figures like Yusuf Mansur and Saptuari Sugiharto in their ‘mathematics of voluntary almsgiving’.

Saptuari has documented the stories of multiple material rewards generated by voluntary almsgiving in his publications. One such account is taken from a courier of *Sedekah Rombongan*, named Karman, who reported on Twitter that he had experienced a 700% reward from his voluntary almsgiving to a religious congregation at an Islamic boarding school in Yogyakarta. On one afternoon, Karman donated IRP 1,000,000 for an Islamic ritual (*mujahadah*) to be held at the boarding school. After this, Karman received a message from a Singaporean buyer who ordered 700 pieces of a batik cloth that he had designed. Every piece of batik was valued as IRP 1,000,000. Explaining Karman's success, Saptuari referred to ‘the verse of the Cow 261 in the Qur’an, stating that “Allah will reward seven hundred multiple rewards for the donors!”’, and he expressed his joy that he was able ‘to witness God's promise’ (Sugiharto and Beatrix 2012, 5).^13^

Making one's economic success transparent on social media is connected to the experience of instant economic gain and thus to a temporal logic of immediacy. This strong association of *sedekah* with a very short time interval between giving and receiving is a phenomenon that can be observed among Muslims in countries other than Indonesia as well. For example, Filippo Osella (2017, 230) found among Muslims in Sri Lanka that ‘Sadaqa … was considered to be akin to an investment that leads to immediate returns: what is given comes back increased manifold’.^14^ And this is exactly what makes *sedekah* so attractive: it not only furthers the accelerated proliferation of wealth, but also fits the temporal logic of social media as media of immediacy.

## Concluding remarks

In this article, we have shown how Islamic charities participate as major non-state actors in Indonesia's field of social welfare. We opted for a genealogical approach paying heed to early twentieth-century debates about the practices of Islamic charity. This approach revealed that, in the late colonial era, although Islamic organizations and their charity initiatives achieved recognition from the colonial authorities, the relationships between state and non-state actors were characterized by a high degree of autonomy. The critique that was launched by reformist Muslims was thus not directed toward state intervention but toward local *zakat* practices that, according to them, had insufficient or no impact on improving the plight of the poor. They argued for the professionalization of managing *zakat* funds that should be used for social welfare programmes. By doing this, reformist Muslims introduced a discourse that was later adopted and further developed by Muslim intellectuals in Suharto's New Order. The largest difference in relation to the colonial era, however, was that this time the state discovered *zakat* as a possible source of funds that could be used to support its development programmes. The response of Muslim intellectuals was varied, and while some understandings of Islamic charity emulated the regime's development discourse, other interpretations broke new ground by introducing the notion of social justice into discussions about *zakat* and development.

These discussions, such as about the possibility to define regular income as wealth that should be subject to *zakat* payments, also reflected the continuing growth of Indonesia's Muslim middle class. Whereas *zakat* became increasingly conceptualized as a modern-day tax, Muslim intellectuals remained cautious when it came to the question of whether (or to what extent) the state should be involved in the collection and distribution of these funds. It thus does not come as a surprise that the most popular *zakat* collection initiatives in the late New Order came from non-state actors, such as the Dompet Dhuafa Foundation, that also expanded their activities and developed a variety of social welfare programmes. It is also noteworthy that this foundation has its origin in an Islamic newspaper initiative, which indicates – already during the late Suharto era – a high degree of interdependence between charity work and its mediation.

In the post-Suharto period, this trend of non-state initiatives continued and even intensified, with a plurality of new actors entering the field of Islamic charities. It also went hand in hand with the emergence of new Islamic authorities that conceptualized the pious Muslim self not only as a reliable payer of *zakat* but also as a disciplined worker and successful entrepreneur. Accordingly, Islamic charity was no longer primarily associated with social welfare and social justice but increasingly also with economic gain. At the same time, *sedekah* as a form of voluntary donation, which is not restricted to a particular share of one's income but potentially unlimited, became more and more popular. The rise of *sedekah* is closely connected with the new Islamic figures who like to propagate it, as well as with the acceleration that Islamic charities have undergone in the last decades, particularly after the introduction of social media. *Sedekah* as it is interpreted in Indonesia today by these popular Islamic figures embodies the idea of instant return, and as such, social media represent the perfect tool to mediate this sense of immediacy, connecting the Islamic charity with the donor, and uniting both of them with the realms of the profane and the divine.

Compared to older forms of Islamic charity, these *sedekah* initiatives operate through a temporal logic of acceleration that finds its expression in an ideology of efficiency and transparency (reminiscent of neoliberal ideas that have been globalized in the last decades), and is demonstrated ‘in action’ on social media. The temporalities implied in the idea of *sedekah* as accelerated return, and in the idea of immediacy as facilitated by social media, converge in the practices of the organizers of these new Islamic charities and their donors. We see here a significant shift away from both late colonial and post-colonial reformist conceptualizations of Islamic charity which envisioned, albeit in different ways, a better future in this world (and, of course, beneficial effects for facing the hereafter as well). This future-orientation and its teleological implications can hardly be discerned in initiatives like *Sedekah Rombongan* that are concerned with accelerating their aid and its mediation, which is appealing to donors that are not only demanding a quick, unbureaucratic conversion of their donations into concrete help, but also an immediate material and spiritual return of their ‘investment’. In view of their emphasis on the here and now, in which this acceleration takes place, these new *sedekah* initiatives give the impression that they are indeed so fast that they leave not only the state but also other Islamic charities behind.

## Footnotes

1 When we were working on this article, on 28 September 2018, Indonesia was hit by another devastating earthquake accompanied by a tsunami in Central Sulawesi. In the days afterwards, the issue of immediate help that was badly needed and the inability of the state to sufficiently provide aid and assistance was widely discussed on Indonesian media.

2 ‘al-Maun’ is one of the verses of the Qur’an that highlights the importance of caring for orphans and the poor.

3 For the problem of mismanagement of *zakat* funds by state institutions and the mistrust this evokes, see also Schaeublin (2014, 25).

4 These figures refer to his following in October 2018.

5 Interview with Efendi Wahyu, branch manager of PPPA Darul Qur’an Yogyakarta, 11 January 2013. See also Kailani (2015).

6 www.saptuari.blogspot.com

7 Interview with Saptuari Sugiharto, 28 March 2013.

8 Since 2010, social media platforms have significantly facilitated the emergence of Islamic charity communities in many parts of Indonesia. In addition to *Sedekah Rombongan*, *Makelar Sedekah* and *Simpul Sedekah* are popular examples.

9 http://www.sedekahrombongan.com/apa-itu-sedekahrombongan (accessed 12 June 2018).

10 Interview with Saptuari Sugiharto, 28 March 2013. However, when *Sedekah Rombongan* collected donations amounting to more than 50 billion rupiah, it had to deal with the Financial Services Authority (Otoritas Jasa Keuangan) by reporting their financial management according to public accounting standards. *Sedekah Rombongan* then started to recruit administrative staff to manage and report their donations (Azis 2018).

11 Interview with Karman and Faishol, two *Sedekah Rombongan* activists, 29 March 2013.

12 http://www.sedekahrombongan.com in the rubric of testimonies (accessed 12 May 2018).

13 However, such a display of economic success in connection with pious deeds comprises ambivalence, as Muslims can be easily accused of committing *riya’*, that is showing off one's piety, which is strongly discouraged and interpreted by some Islamic scholars as a grave sin (Husein and Slama 2018, 86–90). There is thus also a downside to online charity when social media as a medium of transparency apparently reveal undesired, sinful behaviour. Yet such discourses on *riya’* do not seem to significantly reduce the popularity of *sedekah* among Indonesian middle-class Muslims.

14 With ‘sadaqa’, Osella follows the standard transliteration of the Arabic term, whereas in Indonesia the word ‘sedekah’ has become part of the national language Bahasa Indonesia. It is worth mentioning here that a similar practice is also popular among members of charismatic Christian congregations. The followers are encouraged to practice tithing and giving as a way to reach prosperity. Giving is assumed to ‘seed faith’ that will be returned by God in manifold ways (Coleman 2004).
